# Criticality Maximizes Complexity in Neural Tissue

**DOI:** 10.3389/fphys.2016.00425

**Published:** 2016-09-27

**Authors:** Nicholas M. Timme, Najja J. Marshall, Nicholas Bennett, Monica Ripp, Edward Lautzenhiser, John M. Beggs

**Affiliations:** ^1^Department of Psychology, Indiana University - Purdue University IndianapolisIndianapolis, IN, USA; ^2^Department of Neuroscience, Columbia UniversityNew York, NY, USA; ^3^Department of Physics, Indiana UniversityBloomington, IN, USA; ^4^Department of Physics, Syracuse UniversitySyracuse, NY, USA; ^5^Biocomplexity Institute, Indiana UniversityBloomington, IN, USA

**Keywords:** neural criticality, neural complexity, neural avalanche, complex system, power law, shape collapse, information theory

## Abstract

The analysis of neural systems leverages tools from many different fields. Drawing on techniques from the study of critical phenomena in statistical mechanics, several studies have reported signatures of criticality in neural systems, including power-law distributions, shape collapses, and optimized quantities under tuning. Independently, neural complexity—an information theoretic measure—has been introduced in an effort to quantify the strength of correlations across multiple scales in a neural system. This measure represents an important tool in complex systems research because it allows for the quantification of the complexity of a neural system. In this analysis, we studied the relationships between neural complexity and criticality in neural culture data. We analyzed neural avalanches in 435 recordings from dissociated hippocampal cultures produced from rats, as well as neural avalanches from a cortical branching model. We utilized recently developed maximum likelihood estimation power-law fitting methods that account for doubly truncated power-laws, an automated shape collapse algorithm, and neural complexity and branching ratio calculation methods that account for sub-sampling, all of which are implemented in the freely available Neural Complexity and Criticality MATLAB toolbox. We found evidence that neural systems operate at or near a critical point and that neural complexity is optimized in these neural systems at or near the critical point. Surprisingly, we found evidence that complexity in neural systems is dependent upon avalanche profiles and neuron firing rate, but not precise spiking relationships between neurons. In order to facilitate future research, we made all of the culture data utilized in this analysis freely available online.

## 1. Introduction

Interest in the study of complex systems has undergone rapid growth. The principal aim of this field is to seek general frameworks or overarching rules to explain complex behavior in systems that appear to be widely varied (Bar-Yam, 1997; Haken, 2006). The brain has been a subject of considerable treatment under the complex systems paradigm via the analysis of brain networks (e.g., Bullmore and Sporns, 2009). Two important steps for analyzing the brain from a complex systems perspective are devising a metric for quantifying complexity and describing its dependency on changes in the system in such a way that is amenable to comparisons across systems. Though designed for different purposes, neural complexity (Tononi et al., 1994) is an ideal metric of complexity in the brain. Neural complexity is an information theoretic measure that quantifies the strength of correlations across all scales in a neural system. Because information theoretic measures are generally model independent, neural complexity can be applied to any type of system that involves many interacting components and it can be used to quantify how various perturbations to neural systems affect their complexity.

Concurrently, the field of criticality in neural systems has received extensive treatment, primarily via studies of cotemporal sequences of neural activity, otherwise known as “neural avalanches” (Beggs and Plenz, 2003, 2004; Beggs and Timme, 2012). Many recent studies have produced evidence that suggests neural systems are poised at or near a critical point (Beggs and Plenz, 2003; Petermann et al., 2006; Mazzoni et al., 2007; Gireesh and Plenz, 2008; Pasquale et al., 2008; Hahn et al., 2010; Friedman et al., 2012; Priesemann et al., 2013; Lombardi et al., 2014, 2016; Priesemann et al., 2014; Williams-Garcia et al., 2014; Shew et al., 2015). Furthermore, many studies (see Beggs, 2008; Chialvo, 2010; Beggs and Timme, 2012 for reviews) have found important implications for the brain if it is indeed operating at or near a critical point, such as optimal communication (Beggs and Plenz, 2003; Bertschinger and Natschlager, 2004; Ramo et al., 2007; Tanaka et al., 2009; Shew et al., 2011), information storage (Socolar and Kauffman, 2003; Kauffman et al., 2004; Haldeman and Beggs, 2005), computational power (Bertschinger and Natschlager, 2004), dynamic range (Kinouchi and Copelli, 2006; Shew et al., 2009), and phase sychrony (Yang et al., 2012). A great deal of research on criticality in neural systems has focused on the existence of power-laws in neural data (see Beggs and Plenz, 2003; Priesemann et al., 2009; Shew et al., 2009; Klaus et al., 2011; Ribeiro et al., 2014 as examples, Clauset et al., 2009; Touboul and Destexhe, 2010; Dehghani et al., 2012 for critiques, and Beggs and Timme, 2012 for a review), though recent research has expanded the number analysis techniques (Beggs and Timme, 2012) to include shape collapse (Friedman et al., 2012; Priesemann et al., 2013), susceptibility (Williams-Garcia et al., 2014), and tuning through criticality (Shew et al., 2009, 2011). In this work, we included and improved upon many of these analyses.

In this article, we present the first study relating a measure of complexity to criticality in a neural system. This analysis is important because it provides a strong link between these two methods of conceptualizing the brain. Though research in complexity and criticality are motivated by different questions, we hypothesized that complexity and criticality are related in neural systems. It is important to note, however, that their relationship is not trivial. Indeed, as we will discuss below, neural complexity is not dynamic, while the criticality analyses we will apply are dynamic. Neural complexity is dependent on the distribution of states (i.e., patterns of spiking neurons in a time bin) observed in the data without regard to the time ordering of those states. Conversely, neural criticality analyses explicitly rely on time ordered network states (i.e., patterns of spiking neurons across multiple adjacent time bins).

To study the relationship between complexity and criticality in neural systems, we chose to analyze action potential data from dissociated hippocampal cultures from rats grown on large multielectrode arrays. We recorded neural activity from these cultures through the first 5 weeks of development. We chose to use this system because it allowed for the relatively easy collection of large amounts of data (435 recordings including nearly 40,000 neurons) and because similar cultures were previously found to produce critical or near-critical behavior (Tetzlaff et al., 2010). In analyzing the data, we used recently introduced techniques, including: a maximum likelihood estimation (MLE) fitting method for doubly truncated discrete power-laws (Marshall et al., 2016), which allowed us to account for sampling and finite size effects in measuring power-laws (Clauset et al., 2009; Touboul and Destexhe, 2010; Dehghani et al., 2012); an automated method for performing and measuring shape collapses (Marshall et al., 2016), which represented a significant improvement in methodology over previous manual shape collapses analyses (Friedman et al., 2012); methods to account for sub-sampling in complexity calculations, thus improving the accuracy of these calculations in large systems of neural sources; and a branching ratio calculation technique designed to account for sub-sampling (Wilting and Priesemann, 2016).

Using our large data set and advanced analysis tools, we found the following results: (1) In a critical model, we found that complexity peaked near the critical point for the model. (2) In real data, we found critical exponents that agreed with previous studies and expected values. Also, we produced results from shape collapse, power-law fitting, and susceptibility analyses that were not preserved under randomization. In addition, after accounting for sub-sampling we found the real data produced branching ratio values near 1 for the vast majority of the recordings. These results are consistent with the hypothesis that the neural system is operating near a critical point. (3) In the real data, complexity was also not preserved under randomization, indicating that complexity tracks well with other well established methods of assessing critical systems. (4) Surprisingly, we found that complexity in neural systems was primarily dependent upon neuron firing rate and avalanche profiles, not precise spiking relationships between neurons. Taken together, these results are evidence that neural systems operate at or near a critical point and that complexity is also optimized in these neural systems at or near the critical point. (5) In order to facilitate research in this area and others, we have made the culture data freely available online via the CRCNS data sharing initiative (Timme et al., 2016b).

## 2. Materials and methods

### 2.1. Ethics statement

All neural tissue samples from animals were prepared according to guidelines from the National Institutes of Health and all animal procedures were approved by the Indiana University Animal Care and Use Committee (Protocol: 11-041).

### 2.2. Neural cultures and spike sorting

**Figure 1:**
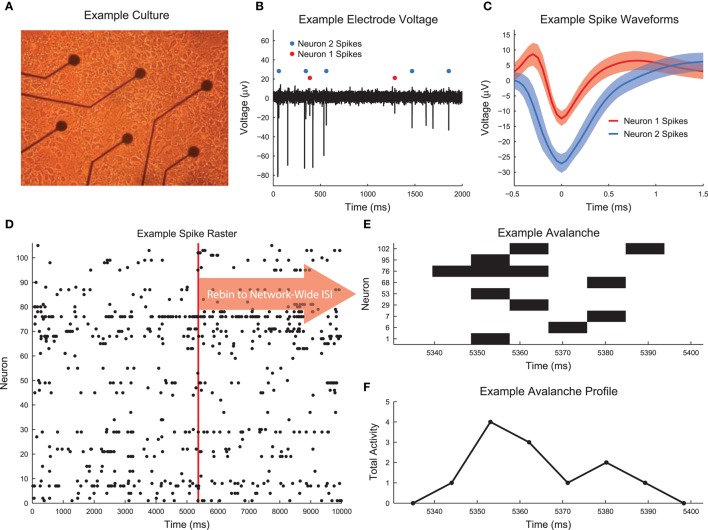
**Culture recording, spike sorting, and avalanche detection**. **(A)** Image of example low density dissociated hippocampal culture plated on the electrode array (50,000 cells, DIV 6). Low density culture produced for testing and imaging purposes. High density cultures (as used in the analysis) were difficult to image due to overlapping cell structure. **(B)** Example voltage recording from a culture that was utilized in the analysis. Spike sorting identified two neurons. **(C)** Average spike waveforms for neurons 1 and 2 from **(B)**. Solid line represents mean voltage and fringe represents one standard deviation. **(D)** A segment of the spike raster for all electrodes in the same culture as the electrode shown in **(B)**. **(E)** Example neuronal avalanche. Once spikes were found for all electrodes with a temporal resolution of 0.05 ms, the data were rebinned to the mean network-wide interspike interval (ISI). Adjacent periods of activity were then identified as avalanches. This avalanche corresponds to the red vertical line in **(D)**. This avalanche was duration 6 (6 time bins long) and size 12 (12 total neuron activations). **(F)** Avalanche profile for the example avalanche shown in **(E)**.

**Figure 2:**
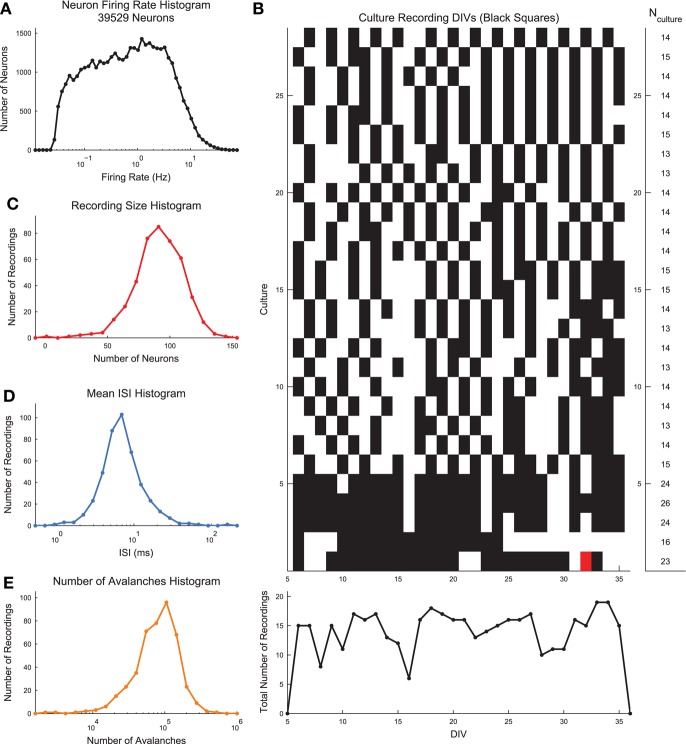
**Basic dissociated culture properties**. **(A)** Neuron firing rate histogram. The histogram does not appear to be log-normal, though firing rates span multiple orders of magnitude and this distribution could result from the sum of multiple log-normal distributions and low firing rate bias in spike sorting. **(B)** Recording DIVs (black squares) for all cultures. White squares indicate no recording was performed. Red squares indicate the recordings that were excluded from the analysis due to too few avalanches. All other recordings were fully utilized in the analysis. **(C)** Histogram of number of recorded neurons across all recordings. **(D)** Histogram of mean network-wide interspike interval (ISI) across all recordings. Each recording was rebinned to the mean ISI. **(E)** Histogram of number of observed avalanches in each recording after rebinning to ISI.

Dissociated hippocampal cultures were produced from rats using the procedures detailed in Hales et al. (2010). Briefly, timed pregnant female rats (Sprague-Dawley from Harlan Laboratories) were euthanized using CO_2_. Embryonic day 18 embryos were removed. Embryonic tissue was used to facilitate the creation of a connected network of neurons following dissociation and plating. The hippocampi of each embyro were extracted and combined from all embryos. The neural tissue was then dissociated and plated on Multichannel Systems 60 electrode arrays (8 X 8 square array with corners removed, 200 μm electrode spacing, 30 μm electrode diameter). We plated cultures with a density of 10,000 cells per μL and we plated a total of approximately 200,000 cells per culture. See Figure 1A for an image of an example low density culture. We recorded from the cultures for days *in vitro* (DIV) 6 through 35. See Figure 2B for a list of the cultures and recording DIV (total number of recordings: 435). We did not record from the cultures for the first five DIV because activity was not generally stable during those DIV (Wagenaar et al., 2006). We analyzed the first 59 min of each recording, conducted at a sampling rate of 20 kHz.

For each recording, we used the wave_Clus spike sorting algorithm to identify individual neuron action potentials (Quiroga et al., 2004). Briefly, the wave_Clus algorithm firsts identified putative neuron action potentials (spikes) using a negative voltage threshold (5 standard deviations). The spike waveforms were then wavelet transformed and the 10 most non-normally distributed wavelet coefficients were then clustered using superparamagnetic clustering. This clustering algorithm utilized an annealing process and was capable of clustering non-circular groupings of points in wavelet coefficient space. All spike sorting results were manually checked using a custom GUI written in MATLAB for this analysis. The original wave_Clus algorithm included a detector dead time of 1.5 ms following a detected action potential to enforce refractory period requirements. This dead time was removed in our analysis because we found it biased detection of spikes from multiple neurons. Given that all results were manually checked and erroneous spikes were removed, we feel the removal of the dead time was appropriate. See Figure 1B for an example voltage trace segment with detected neuron action potentials. See Figure 1C for example spike waveforms for this example electrode. The spike sorting algorithm yielded spike times at a resolution of 20 kHz for all found neurons in the recordings. See Figure 2A for the neuron firing rate histogram and Figure 2C for the histogram of number of neurons found in each recording (total number of neurons across all recordings: 39,529). Most recordings yielded approximately 100 spike sorted neurons and firing rates ranged from roughly 0.05–20 Hz. Note that we were not able to record from all approximately 200,000 plated neurons due to the physical limitations of the electrode array (e.g., number of electrodes and area covered by the array) and because not all plated neurons survive and are integrated into the network. All of the spike sorted recordings are freely available via the CRCNS data sharing initiative (Timme et al., 2016b).

### 2.3. Neural avalanches

Neural avalanches were identified as sequences of time bins during which at least one neuron spiked (Figures 1D–F). Prior to neural avalanche detection, the spiking activity of each recording was rebinned to the average network-wide interspike interval for that recording (i.e., the interspike interval was calculated considering all spikes for all neurons). We used the mean network-wide ISI because it provided a convenient estimate for the characteristic time scale of the system. In addition to being easy to calculate, the ISI intuitively scales with number of neurons and neuron firing rate. This rebinning procedure was necessary because very small bins would result in only very small avalanches, while very large bins would result in only very large avalanches. Most recordings had ISIs of around 7 ms, though some data sets showed much higher or lower ISIs (Figure 2D). We feel this result highlights the need for some type of adaptive method for setting the bin size (e.g., the ISI method we employed), rather than simply setting a uniform bin size for all data sets. Some data sets possessed larger avalanches and/or faster spiking neurons in avalanches, requiring smaller bins, while other data sets had smaller avalanches and/or slower spiking neurons in avalanches, requiring larger bins. Following the rebinning procedure and avalanche detection, most recordings contained around 10,000 avalanches (Figure 2E).

### 2.4. Models

We employed two types of branching models in this analysis (Haldeman and Beggs, 2005; Williams-Garcia et al., 2014). These models were capable of producing neural avalanches that could be analyzed using all of the methods used to analyze the real data. First, we used a simple branching process (or Bethe lattice) model, consisting of layers of feed-forward neurons with no recurrent connections. Within a layer, each neuron sent two connections to neurons in the next layer. Each neuron in a given layer received only one connection. Therefore, each layer contained twice as many neurons as the previous layer. For each run of the model, the single neuron in the first layer was activated. Activity was spread from active neurons with a likelihood of *p*_*trans*_ = 0.5. This transmission probability resulted in a model tuned to its critical point. The model could be run infinitely, but practical limitations meant that we were forced to stop activity at the 10^4^ layer. We generated one version of this model to demonstrate the difference between complexity and criticality (see Section 3.1).

Second, we used a cortical branching (CB) model that contained 100 neurons arranged in a square lattice with periodic boundary conditions (i.e., a torus). The model utilized a spontaneous firing probability to randomly create activity in the network. At each time step, each neuron had a $p_{spont}=10^{-4}$ likelihood to activate spontaneously. Activity in the network propagated via interactions between a neuron and its four nearest neighbors. If a given neuron was active at time *t*, there was a *p*_*trans*_ likelihood that it would cause a given one of its neighbors to be active at time *t* + 1. We tested values of *p*_*trans*_ on the interval 0.2 ≤ *p*_*trans*_ ≤ 0.3 with increments of 0.05. We generated 30 example models for each unique *p*_*trans*_ value. Each cortical branching model was run for 3^*^10^5^ time steps. In a version of the cortical branching model with infinite neurons, the critical point is near *p*_*trans*_ = 0.25. So, we expected that the near-critical point for the finite model we employed would also occur for *p*_*trans*_ ≈ 0.25 (see Section 2.10).

In addition to the branching models, we also used a simplified model to generate spiking activity with varying degrees of complexity. This model consisted of 12 neurons arranged in a feedforward chain (i.e., neuron 1 could influence neuron 2, but not vice versa). For each run of this model, the first neuron in the chain was either active or inactive (alternating between runs of the model). The spiking state of the *ith* neuron (call it *a*_*i*_) was coupled to the spiking state of the (*i* + 1)^th^ neuron (call it *a*_*i*+1_) using a parameter *c* such that *p*(*a*_*i*+1_ = *a*_*i*_) = 0.5(1 + *c*) and *p*(*a*_*i*+1_ ≠ *a*_*i*_) = 0.5(1 − *c*). Therefore, the system produced totally random data for *c* = 0 and totally ordered data for *c* = 1. In this analysis, we utilized values of *c* = 0, 0.8, 1 to probe different levels of complexity.

### 2.5. Data randomization methods

We utilized several randomization methods in this analysis to probe the importance of different qualities of the data (e.g., neuron firing rate, avalanche profile, etc.) in the culture data and in the cortical branching model data. See Table 1 for a description of which features of the data are preserved under each type of randomization. In the culture data, all randomization was performed after rebinning. Because of this and explicit requirements in the randomization algorithms, we insured that the spike count for each neuron did not change as a result of randomization in all algorithms except shuffling. We found that accidentally failing to maintain spike count can cause erroneous results. No rebinning was required in the cortical branching models because the data were already binned at the appropriate time scale when they were produced.

**Table 1:** **List of data features preserved under randomization for each randomization method**.

**Randomization method**	**Firing rate**	**ISI**	**Avalanche profile**
Jitter	Yes	No^a^	No^b^
Wrap	Yes	Yes^c^	No
Poisson	Yes	No	No
Swap	Yes	No	Yes
Shuffling	No	No	Yes

a *Because spike jittering symmetrically randomized spikes, it did not precisely preserve the interspike intervals for each neuron, but the interspike interval distribution for each neuron was approximately preserved*.

b *As with the interspike interval, spike jittering did not precisely preserve the avalanche profiles, but it did so approximately, especially for small jitter*.

c *Wrapping altered only one interspike interval (at the random cut point)*.

We utilized five randomization methods in this analysis. First, we jittered the data by randomly moving spike times earlier or later in the spike trains. We determined the amount by which the spike would move using a normal distribution centered on the original spike time. The standard deviation of the normal distribution was 1, 10, or 100 time bins. We insured that this process did not change the neuron firing rate (i.e., by accidentally jittering two spikes into one bin). But, this method did alter the neuron interspike intervals (ISIs) and the avalanche profiles. That said, the ISIs were similar before and after jittering because the jittering process was symmetric (i.e., there was an equal likelihood of moving spikes forward in time by some Δ*t* as there was to move spikes backward in time by Δ*t*). Furthermore, avalanche profiles were altered more severely with larger jitter size.

Second, we wrapped the data by cutting each neuron spike train at a random unique point and swapping the remaining halves. Note that this process preserved the neuron firing rate and nearly perfectly preserved the ISIs. One ISI was altered (at the cut point), but all other ISIs were preserved. Also, wrapping severely altered avalanche profiles by decorrelating the neurons.

Third, we Poisson randomized the data by randomly placing a neuron's spikes with equal likelihood at all time points. This process essentially converted each neuron into a random Poisson process with firing rate matching the original data. Therefore, this method preserved neuron firing rate, but greatly altered the ISIs and the avalanche profiles. In one portion of the analysis, we slightly altered the Poisson randomization procedure by moving whole network states for each individual time bin instead of treating each neuron separately (see Section 3.1).

Fourth, we spike swapped the data by trading pairs of spikes between neurons. This process consisted of finding a pair of spikes from two neurons such that the neuron identities for the spikes could be traded without deleting spikes (i.e., neuron A spikes at *t*_1_, but not *t*_2_, while neuron B spikes at *t*_2_, but not *t*_1_) and then trading the neuron identities for those spikes. Every spike was swapped at least once if a possible. Note that spike swapping preserved neuron firing rates and the avalanche profiles because the total number of spikes at each time bin was not altered. However, spike swapping did not preserve neuron ISI. By moving a neuron's spikes from one time bin to another, spike swapping disrupted spiking relationships between neurons.

Fifth, we randomized the neuron identities for spikes at every time step. We referred to this process as “neuron shuffling” or “shuffling.” For instance, if five neurons spike at a given time step, we randomly assigned those spikes across all neurons with equal likelihood. This randomization was carried out at each time step in the recording. Note that this process did not preserve neuron firing rate (unlike all other methods), nor did it preserve the neuron ISIs. However, because it preserved the total number of active neurons at each time step, it did preserve the avalanche properties.

### 2.6. Critical exponents

The study of critical phenomena in statistical mechanics provides concepts and notation that can be readily applied to neural avalanches (Sethna et al., 2001; Friedman et al., 2012). A neural avalanche is characterized by its duration (number of contiguous time bins in which at least one neuron fired) and size (total number of active neurons). If a neural network operates near a critical point, then the size distribution (*f*_*s*_(*S*)), duration distribution (*f*_*d*_(*T*)), and average size given duration data (〈*S*〉(*T*)) of its avalanches can be fit to power laws (Equations 1–3).

(1) $$f_{s}(S)\;\propto S^{-\tau }$$

(2) $$f_{d}(T)\;\propto T^{-\alpha }$$

(3) $$\langle S\rangle (T)\;\propto T^{1/\sigma \nu z}$$

In Equations (1–3), *S* is the size of an avalanche and *T* is the duration of an avalanche. The power-law exponents τ, α, and 1/σ*νz* are critical exponents of the system. They are model independent and identical for all systems in the same universality class (Sethna et al., 2001; Friedman et al., 2012). Furthermore, the critical exponents themselves are related via (Equation 4) (Friedman et al., 2012).

(4) $$\begin{array}{l} \frac{\alpha -1}{\tau -1}=\frac{1}{\sigma \nu z} \end{array}$$

Testing this relationship has been proposed as an important evaluation of the critical state of the system (Friedman et al., 2012).

### 2.7. Power-law fitting

The subject of power-law fitting in general (Burroughs and Tebbens, 2001; Goldstein et al., 2004; Perline, 2005; White et al., 2008; Clauset et al., 2009; Priesemann et al., 2009; Holden and Rajaraman, 2012; Deluca and Corral, 2013) and power-law fitting in analyses of neural criticality in particular (Priesemann et al., 2009; Touboul and Destexhe, 2010; Klaus et al., 2011; Dehghani et al., 2012; Alstott et al., 2014; Ribeiro et al., 2014; Yu et al., 2014; Touboul and Destexhe, 2015) have received a great deal of attention in the literature. We recently introduced improved power-law fitting methodologies that account for doubly truncated data, including software support via the MATLAB NCC toolbox (Marshall et al., 2016). These improvements built upon previous advances (Clauset et al., 2009; Deluca and Corral, 2013) by utilizing an automated maximum likelihood estimation (MLE) technique to detect power law portions of data histograms. This method can automatically control for data sets with sub-sampling bias in the tail of a power-law distribution. Given the fact that neural avalanche recordings are highly sub-sampled (i.e., a very small percentage of neurons are recorded), this improvement is especially important in power-law analyses of neural avalanches. We will now briefly review this methodology (see Marshall et al., 2016 for a complete discussion and demonstration of the fitting routine).

In neural avalanche analyses, we will be interested in fitting the distributions of avalanche sizes and durations (see Section 2.3). Prior to fitting the distributions, we applied cuts to the data. For a given type of distribution (size or duration), we removed avalanches with sizes or durations less than 4 as well as data for which less than 20 avalanches of that size or duration were observed. These cuts were imposed in order to consider similar portions of the data in the power-law fit analysis as we considered in the shape collapse analyses (see Section 2.8). Note that because the fitting method can account for doubly truncated data, removing data from the left and right portion of the distribution via these cuts did not bias the fitting as would be the case with methods that do not account for double truncation.

After the application of cuts, we fit the histogram using the doubly truncated discrete power-law MLE. To assess if the power-law fit was acceptable, we used the fit to generate 500 power-law distributed model data sets (with matching numbers of avalanches). We then compared the KS-statistic between (1) the fit and the real data vs. (2) the fit and the power-law model data. If the real data produced a KS-statistic that was less than the KS-statistic found for at least 20% of the power-law models (i.e., *p* ≥ *p*_*thresh*_ = 0.2), we accepted the data as being fit by the truncated power law because the fluctuations of the real data from the power law were similar in the KS-statistic sense to random fluctuations in a perfect power-law model. However, if the converse was true, the truncated power-law hypothesis was rejected. Note that this method was not able to prove the data were generated by a truncated power law (nor could any fitting algorithm), rather it was only able to reject the truncated power-law hypothesis.

If the truncated power-law hypothesis was rejected, we searched for successively smaller ranges of the distributions that could be fit by the truncated power-law using the same methodology discussed above. We defined the ranges logarithmically in terms of decades or orders of magnitude. Once a range was found over which the truncated power-law hypothesis was accepted, we ceased the search. Because the algorithm searched through successively smaller fit ranges, the fit ranges reported by the analysis represent the largest segment of the data that was fit by a truncated power-law.

In addition to fitting the size and duration distributions with truncated power laws, we also wished to fit the values of the average avalanche sizes given duration for each data set. These data are also hypothesized to follow a power law (see Section 2.6). However, unlike the size and duration distributions, the average size given duration plots show power laws with positive exponents. This is expected since long duration avalanches, while less likely than short duration avalanches, are more likely to have a larger size than short duration avalanches. Because the average size given duration data is not a probability distribution, we were unable to fit it using an MLE approach. Instead, we used a simple weighted least squares fitting algorithm via the standard Matlab function lscov. We only fit size given duration data for avalanches that fell in the duration range fit by a truncated power law using the methods discussed above.

### 2.8. Shape collapse

If a neural system is in a critical state, in addition to exhibiting power-law size and duration distributions, the mean temporal profiles of avalanches should be identical across scales (Friedman et al., 2012). In other words, the profiles (Figure 1F) of long duration avalanches should have the same scaled mean shape as short avalanches. This phenomenon is also referred to as “shape collapse.” Specifically, the mean number of spiking neurons (*s*) at time *t* in an avalanche of duration *T* is related to the universal scaling function for the avalanche temporal profile *F* via (Equation 5) (Friedman et al., 2012).

(5) $$\begin{array}{l} s\left(t,T\right)\propto T^{\gamma }F\left(t/T\right) \end{array}$$

In Equation (5), γ is the scaling parameter that controls how much larger in size long duration avalanches are than short duration avalanches. Therefore, if the correct scaling parameter γ is chosen and if the system is close to criticality, when plotted on a scaled duration (i.e., *t*/*T*) avalanches of all durations should produce the same mean profile when scaled via *s*(*t, T*)*T*^−γ^.

Using Equations (3), (5), and (6), it can be shown (Friedman et al., 2012) that 1/σ*νz* (see Equation 3) is related to the shape collapse scaling parameter γ via (Equation 7).

(6) $$\langle S\rangle (T)\;=\int_{0}^{T}s(t,T)dt$$

(7) $$\gamma \;=\frac{1}{\sigma \nu z}-1$$

Because γ can be expressed in terms of the critical exponent 1/σ*νz*, we will only quote 1/σ*νz* in relation to shape collapses throughout this analysis. Therefore, it is possible to measure 1/σ*νz* using both the shape collapse and the average size given duration. The comparison between these values—which should be identical if the system is truly poised at a critical point—can be an important check of the criticality hypothesis.

In this analysis, we used shape collapse methodologies introduced in the NCC Toolbox (Marshall et al., 2016). These techniques improved upon previous manual shape collapse analyses (Friedman et al., 2012; Priesemann et al., 2013) by performing an automated collapse. Briefly, the mean temporal profile of all avalanches with durations longer than 3 and with at least 20 examples were collapsed such that the normalized variance between the mean avalanche shapes was minimized. Note that this method replaced the subjective classification of avalanches as exhibiting shape collapse (Friedman et al., 2012) with a quantitative assessment of the best possible collapse. Furthermore, we quantified the shape of the collapse by fitting the collapsed avalanches with a quadratic polynomial and calculating its average curvature. We did not seek to evaluate if the data exhibited a shape collapse because no quantifiable method exists to assess when a group of avalanches exhibits or does not exhibit shape collapse (though see Shaukat and Thivierge, 2016 for a recent attempt to do so). Rather, we feel that it is more appropriate to apply the shape collapse algorithm and interpret the resulting scaling parameter and curvature.

### 2.9. Complexity

Neural complexity is a measure of the degree to which neurons interact across multiple scales in a neural system. We utilized the original definition of complexity (Tononi et al., 1994), but with several improvements to account for sub-sampling (Marshall et al., 2016). We will now briefly discuss complexity and how it is defined.

We calculated the complexity in a system of *N* spiking neurons (call this system *X*). The entropy of a system of *N* spiking neurons is given by Equation (8) (Cover and Thomas, 2006).

(8) $$\begin{array}{l} H\left(X\right)=-\underset{i}{\sum }p\left(x_{i}\right)\log\left(p\left(x_{i}\right)\right) \end{array}$$

In Equation (8), *x*_*i*_ is a joint state of all *N* neurons at a given time bin (i.e., combinations of spiking and not spiking) and the base of the logarithm is 2 to yield information results in units of bits. In our analysis, the probability of a given joint state of neurons *p*(*x*_*i*_) was found by counting the number of occurrences of a given state throughout a recording and dividing by the total number of states. We assumed the probability distribution *p*(*x*_*i*_) was stationary throughout the recording.

By comparing the joint entropy of a group of neurons to the sum of their individual entropies, it is possible to measure the degree to which the activities of the neurons are coordinated. This measure is referred to as the integration (the integration has previously been referred to as the total correlation Watanabe, 1960; Tononi et al., 1994; Timme et al., 2014a). When considering a subset of neurons, we note the *jth* unique set of *k* neurons as $X_{j}^{k}$. So, $X_{j}^{1}$ would refer to the *jth* neuron alone, but $X_{j}^{3}$ would refer to the *jth* unique set of 3 neurons. Using this notation, the integration of the *jth* set of *k* neurons is given by Equation (9).

(9) $$\begin{array}{l} I\left(X_{j}^{k}\right)=\left(\underset{j^{\prime }\in k}{\sum }H\left(X_{j^{\prime }}^{1}\right)\right)-H\left(X_{j}^{k}\right) \end{array}$$

Using the integration, the complexity is given by Equation (10) (Tononi et al., 1994; van Putten and Stam, 2001).

(10) $$\begin{array}{l} C_{N}\left(X\right)=\frac{1}{N}\overset{N}{\underset{k=2}{\sum }}\left[\left(\frac{k-1}{N-1}\right)I\left(X_{1}^{N}\right)-{\langle I\left(X_{j}^{k}\right)\rangle }_{j}\right] \end{array}$$

In the data analysis, the average subset integration (${\langle I\left(X_{j}^{k}\right)\rangle }_{j}$) was calculated at each possible value of *k* from 2 to *N*. For a given value of *k*, we calculated ${\langle I\left(X_{j}^{k}\right)\rangle }_{j}$ for the lesser of either all possible permutations of neurons or 100 randomly chosen permutations. In Equation (10), $I\left(X_{1}^{N}\right)$ represents the integration of the whole system, thus the first term in the brackets represents a linear approximation for the expected integration across all scales of the system given the total integration.

**Figure 3:**
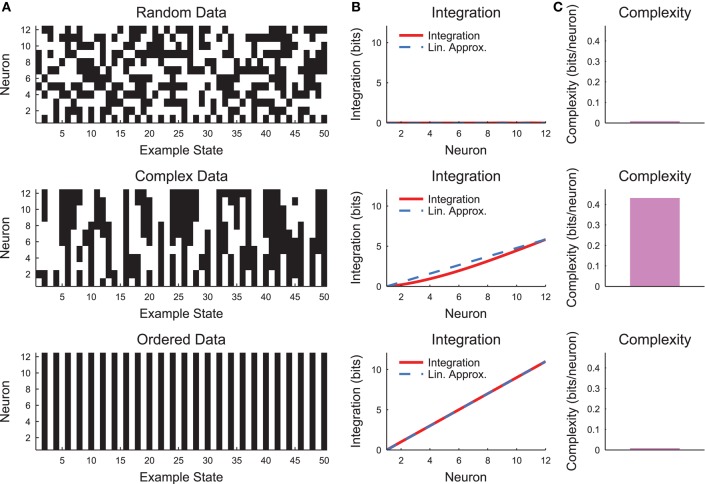
**Neural complexity**. **(A)** Short segments of example spike rasters for three types of chain model data (see Section 2.4, *c* = 0 (random), *c* = 0.8 (complex), *c* = 1 (ordered)). **(B)** Integration curves with linear approximations for different subset sizes. Note that random data shows no integration, while ordered data shows high integration. Complex data shows high integration that varies non-linearly with subset size. **(C)** Complexity values. Only the complex data shows non-zero complexity.

The complexity as expressed in Equation (10) can be difficult to interpret. Therefore, it is helpful to evaluate the complexity in a simple system such as a small chain model (see Section 2.4, Figure 3). Complexity requires some degree of coordinated variability across many scales in the system. In Figure 3, we show three types of models: a random model, a complex model, and an ordered model. The behaviors of the models are apparent from a brief segment of representative spike rasters (Figure 3A). The random data contain no correlations, while the ordered data contain no variability. The complex data show some balance between these states. When the integration curves are plotted (Figure 3B), the random data produce zero integration, while the ordered data produce high integration. However, the complex data produce a non-linear integration curve, suggesting varying correlations across scales and non-zero complexity.

The model used to generate example results for Figure 3 was small and well defined (i.e., the precise joint probability distributions were defined). Conversely, neural data typically include many more variables and the joint probability distribution must be estimated from available observations. This reality of experimental data is likely to lead to state sub-sampling bias (Marshall et al., 2016). Previous analyses have dealt with this issue by making assumptions about the underlying structure of the data (e.g., converting neural signals to Gaussian distributions Tononi et al., 1994). We introduced a sub-sampling detection technique that automatically finds the largest point at which the system can be accurately sampled and calculates a corrected complexity for this smaller subset of neurons (Marshall et al., 2016). In this analysis, we utilized this methodology as implemented in the NCC toolbox (Marshall et al., 2016). Furthermore, unless otherwise noted, we only analyzed the complexity of neural avalanches to avoid biases associated with the number of avalanches in a recording.

### 2.10. Sub-sampling

In order to account for the finite size of the samples and the fact that the culture data samples contained different numbers of neurons, we utilized a sub-sampling method to extrapolate various quantities to systems with infinite size. In this analysis, both the cortical branching model and our culture data represent finite size systems. The cortical branching model contained 100 neurons for which we have a complete record of spiking activity. The culture data contained at most approximately 200,000 neurons (see Section 2.2) for which we have only a small fraction of the complete record of spiking activity (~100 neurons). The impact of undersampling on assessments of criticality have been discussed previously in the literature (Priesemann et al., 2009; Ribeiro et al., 2014). For both types of data, we sub-sampled the measured neurons and calculated various quantities (e.g., size distribution power-law fits). We then plotted the resulting quantities as a function of inverse number of neurons and performed a linear fit using weighted least squares. The y-intercept for this fit then corresponded to an estimate for the given parameter in an infinite or very large system.

**Figure 4:**
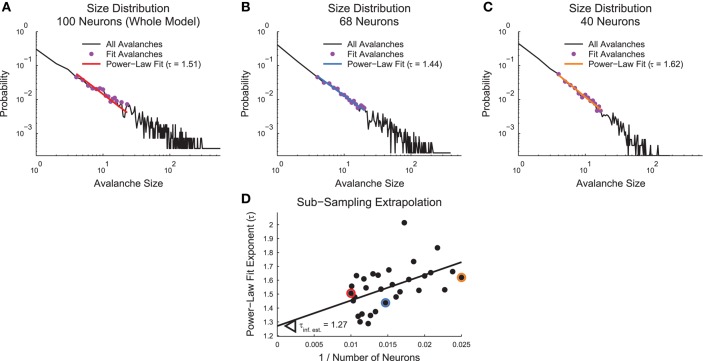
**Sub-sampling algorithm**. **(A–C)** Power-law fits for avalanche size distributions for a full example cortical branching model **(A)**, a sub-sampled version of the same model with 68 neurons **(B)**, and a sub-sampled version of the same model with 40 neurons **(C)**. **(D)** Scatter plot of power-law fit exponents vs. inverse sub-sample size. A weighted linear least squares fit produces an extrapolation of the value for infinite system size at the y-intercept. Highlighted data points correspond to fits from **(A–C)** via color coding.

The specific sub-sampling routine we utilized is as follows (Figure 4): We randomly sub-sampled each system 30 times at evenly spaced systems sizes from 40% of the recorded system size to the full recorded system size. We then calculated the relevant values of interest for the sub-sampled system (e.g., size distribution power-law fits). These analyses were identical to those performed in the whole recorded system as described above with the exception of the power-law MLE fits. We found that the power-law fit search algorithm was unstable under sub-sampling. So, for sub-sampling the power-law MLE fits, we utilized the fit value for all avalanches that survived the minimum size/duration and minimum occurrence cuts. Next, we plotted the values vs. the inverse neuron number for the given sub-sample. We fit these data using a linear weighted least squares fit. The number of neurons for each sub-sample was used as the weight for the fitting. This fitting procedure was applied to all sub-samples in all analyses with the exception of the complexity data from the cortical branching model. We used a different fitting method for these data because they exhibited a discontinuity in the complexity trend. For those data, we fit the 10 largest sub-samples as described above. Progressively smaller sub-samples were added and fit until the newest point produced a fit residual that was larger than the mean residual plus 3 standard deviations of the residuals. Following the fitting, in all cases the y-intercept (i.e., 1/*N* = 0) was interpreted as an estimate for the quantity of interest in an infinite or very large system.

We performed the sub-sampling analysis in non-randomized cortical branching model and culture data. In the cortical branching model data, we performed the sub-sampling analysis for all quantities under study except the branching ratio (see Section 2.11). In the culture data, we applied the sub-sampling analysis to only the critical exponents (see Section 2.6). We limited the sub-sampling analysis in the culture data because we found the sub-sample values for other quantities (e.g., complexity) were not well fit by a linear function and require more advanced fitting techniques.

Note that the sub-sampling method we employed was not finite size scaling (Cardy, 1988). This is because finite size scaling is concerned with evaluating the relationship between the behavior of quantities in finite versions of systems with the behavior of those quantities in the infinite version of those systems. For instance, only an infinite system can exhibit a true critical point where correlation lengths diverge to infinity. In finite systems, the correlation length may show a peak at a temperature near the critical point for the infinite system, but the correlation length will not diverge to infinity and the peak temperature in the finite system will most likely differ from the critical temperature in the infinite system. Finite size scaling provides tools to link finite system behavior with infinite system behavior. This is necessary in practice because some infinite systems are easier to analyze analytically than their finite versions, while, for other systems, the infinite systems are very difficult to analyze analytically, but their finite versions are easy to simulate.

### 2.11. Branching ratio and susceptibility

The branching ratio has previously been used to quantify the stability of neural activity (Haldeman and Beggs, 2005; Wilting and Priesemann, 2016). In its most basic form, the branching ratio is simply the time average number of active neurons at time *t* + 1 (*A*(*t* + 1)) divided by the number of active neurons at time *t* (*A*(*t*)):

(11) $$B_{basic}={\langle \frac{A(t+1)}{A(t)}\rangle }_{t}$$

If a system is near the critical point, the activity in the network will be stable (*B*_*basic*_ ≈ 1). If the system is sub-critical, the activity in the network will die off quickly when it appears (*B*_*basic*_ < 1). If the system is super-critical, the activity in the network will expand quickly (*B*_*basic*_ > 1).

We utilized a recently introduced improvement to the branching ratio calculation methodology that accounts for system sub-sampling (Wilting and Priesemann, 2016). We have added the function brestimate to the NCC MATLAB toolbox to carry out this calculation (Marshall et al., 2016; Timme, 2016) and we will briefly review the methodology here.

At the heart of the method introduced by Wilting and Priesemann is examining activity at various delays (*k*) in the system and observing how the ratio of activity changes with delay. First, we calculated the slope *r*_*k*_ of the linear regression between *A*(*t* + *k*) and *A*(*t*):

(12) $$\begin{array}{l} A\left(t+k\right)=r_{k}*A\left(t\right) \end{array}$$

This fitting was performed using least squares fitting with the standard MATLAB function polyfit. Next, Wilting and Priesemann showed that the slope of these regressions is directly related to the branching ratio via:

(13) $$\begin{array}{l} r_{k}\sim B_{new}^{k} \end{array}$$

We fit the *r*_*k*_ slope values with an exponential function using non-linear least squares fitting with the standard MATLAB function lsqcurvefit to obtain the branching ratio *B*_*new*_.

Because of finite size effects in our cortical branching model and break down in correlation between activity across long delays, we limited delays such that 1 ≤ *k* ≤ 8. We also limited the activity values considered for the linear regression fits to 0 ≤ *A*(*t*) ≤ 5 for the cortical branching model and to 0 ≤ *A*(*t*) ≤ 10 for the culture data. Note that because the branching ratio calculation we used accounted for sub-sampling, we did not apply the sub-sampling correction routine to the branching ratio calculation (see Section 2.10).

We also calculated the susceptibility of the cortical branching model and the cultures (Williams-Garcia et al., 2014):

(14) $$\begin{array}{l} \chi =var\left(\frac{A\left(t\right)}{N_{neurons}}\right) \end{array}$$

The susceptibility quantifies the degree to which fluctuations in the state of each element of a system propagate to its neighbors. If the system is near the critical point, it will show high susceptibility because changes in the network are sustained over long distances. Conversely, if the system is sub-critical or super-critical, fluctuations will either quickly die out (producing consistently low activity) or saturate the network (producing consistently high activity), respectively. In those cases, the effects of activity changes in one element of the network will be minimal and the susceptibility will be small.

## 3. Results

### 3.1. Complexity and criticality are not identical

**Figure 5:**
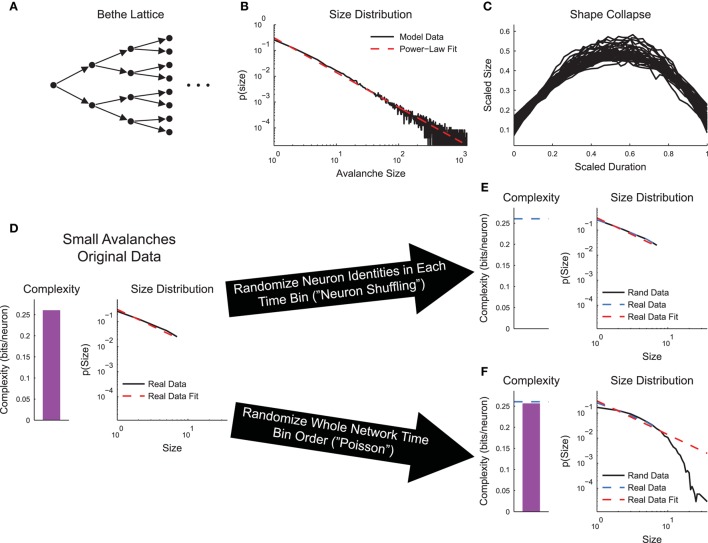
**Neural complexity and criticality are not trivially related**. **(A)** Bethe Lattice or branching model. Activity spread from layer to layer starting at the leftmost neuron. The likelihood to transmit activity along a connection was *p*_*trans*_ = 0.5, yielding a critical model system. **(B)** The avalanche sizes in the model were power-law distributed, as expected. **(C)** The avalanches exhibited shape collapse, as expected. **(D–F)** Analysis of small avalanches (stopped on or before the 8th layer). **(D)** The small avalanches exhibited power-law distributed sizes and non-zero complexity. **(E)** Randomizing the neuron identities (“neuron shuffling”) at each time bin preserved avalanche size distribution, but removed complexity. **(F)** Randomizing the order of the network states (“Poisson” randomization applied to whole network states) preserved complexity, but disrupted the avalanche size distribution.

To ensure that complexity and criticality were not trivially related, we used a simple branching or Bethe Lattice model tuned to the critical point (see Section 2.4, Figure 5A). Using the full model, we found the power-law distributed avalanche sizes (Figure 5B) and shape collapse (Figure 5C). We then limited our analysis to only the avalanches that stopped on or before the 8th layer (“small avalanches”). We performed this truncation because the number of neurons grows exponentially with network layer. This growth severely impacted sub-sampling in the complexity calculation. In the original small avalanches, the sizes of the avalanches were power-law distributed and a non-zero complexity was observed (Figure 5D). We then performed two types of randomizations. First, we randomized the neuron identities in the avalanches (neuron shuffling). This operation preserved the avalanches and their power-law size distributions, but removed the complexity (Figure 5E). Conversely, when we randomized the order of the network states, but preserved neuron spiking states at each individual time bin (Poisson randomization for the whole network state), the complexity was preserved, but the distribution of avalanche size changed dramatically (Figure 5F). This difference in behavior under randomization highlights the differences between criticality and complexity analyses. Complexity only focuses on instantaneous correlations between specific neurons, whereas criticality analyses only focus on the time order of the total number of active neurons.

### 3.2. Cortical branching model

**Figure 6:**
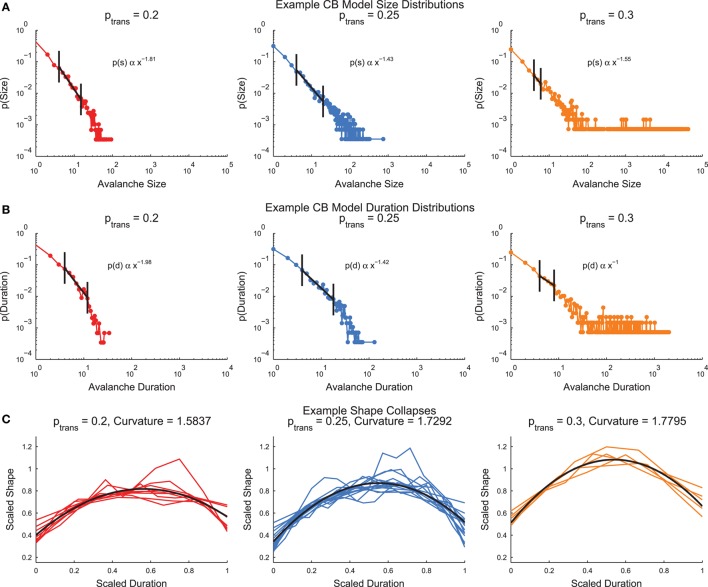
**Example cortical branching model distributions and shape collapse**. **(A)** Size distributions for three example models with low (left), near critical (center), and high (right) transmission probabilities. **(B)** Duration distributions for three example models with low (left), near critical (center), and high (right) transmission probabilities. In **(A,B)**, note that the low transmission probability distributions curve downwards, while the high transmission probability distributions curve upwards. This behavior is indicative of sub-critical and super-critical systems. Automatically detected truncated fit regions marked in black (see Section 2.7) **(C)** Example shape collapses. Note that all three transmission probabilities qualitatively appear to exhibit shape collapse. Quadratic fit of shape collapse shown in black (see Section 2.8).

The cortical branching model (see Section 2.4) produced neural avalanches that were similar to the culture data (see Section 3.3 below). The model produced size and duration distributions that were fit by truncated power-laws after applying minimum size/duration and occurrence cuts (Figures 6A,B, see Section 2.7). In the size and duration distributions, note that the low transmission probability models produced distributions that curved downwards, whereas the high transmission probability models produced distributions that curved upwards. The model with a transmission probability near the infinite system critical transmission probability (*p*_*trans*_ = 0.25) produced the straightest size and duration distributions. This behavior is typical of a sub-critical (*p*_*trans*_ < *p*_*crit*_), critical (*p*_*trans*_ ≈ *p*_*crit*_), and super-critical (*p*_*trans*_ > *p*_*crit*_) system. The cortical branching model also produced shape collapses (Figure 6C, see Section 2.8). Interestingly, the shape collapses qualitatively appear to be of high quality for all transmission probabilities.

**Figure 7:**
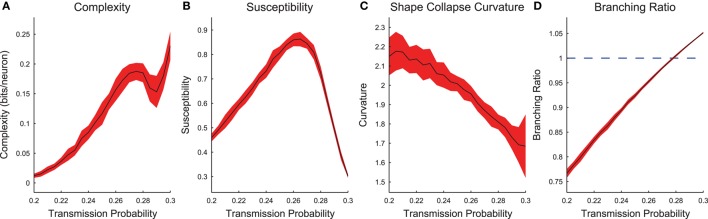
**Cortical branching models exhibit peaks in complexity and susceptibility, and branching ratios close to 1 near the infinite model critical point**. **(A,B)** Complexity and susceptibility peaked near *p*_*trans*_ = 0.25 indicating that both were maximized in critical systems. Note that complexity **(A)** also showed divergent behavior for high transmission probabilities. This behavior was due to finite recording length bias (Figure S2). **(C)** Shape collapse curvature did not peak near *p*_*trans*_ = 0.25, though this was not expected. This indicates that low transmission probability avalanches were more curved and high transmission probability avalanches were more flat. **(D)** The branching ratios (Equation 13) of the networks were near 1 (critical state) near the transmission probabilities that showed peak complexity **(A)** and susceptibility **(C)**. In all sub-figures, the black line represents the average value and the red fringe represents ± one standard deviation.

Now we will examine the combined behavior of all cortical branching models across transmission probabilities (0.2 ≤ *p*_*trans*_ ≤ 0.3) after sub-sampling (see Figure S1 for example sub-sampling fits). When we plotted the complexity and susceptibility vs. the transmission probability, we found both peaked near *p*_*trans*_ ≈ 0.265 (Figures 7A,B). Given that complexity and susceptibility showed maxima near the critical transmission probability for an infinite system (*p*_*trans*_ ≈ 0.25), and susceptibility has been shown to be maximized near criticality (Williams-Garcia et al., 2014), this result is strong evidence that neural complexity is also maximized near criticality. Furthermore, the branching ratio was found to be closest to 1 near this same transmission probability (Figure 7D). A branching ratio of 1 indicates sustained levels of activity necessary for a system to be operating at a critical point, as opposed to branching ratios below one which indicate a system is sub-critical or above one which indicate a system is super-critical.

In addition to the peak in the complexity near *p*_*trans*_ ≈ 0.265 (Figure 7A), we also found that the complexity was increasing near the boundary for the tested range of transmission probabilities (i.e., near *p*_*trans*_ = 0.3). This secondary peak or divergence was due to a bias in the complexity calculation. We found that longer recordings produced higher complexity values (Figure S2). Because we analyzed only the avalanches and higher transmission probabilities produce longer avalanches, high transmission probability models possessed effectively longer recordings. When this effect was controlled by analyzing all time bins in a recording, the secondary peak decreased with longer recordings relative to the primary peak (Figure S2).

We found that the shape collapse curvature did not peak near the critical point (Figure 7C). However, the curvature is not an indication of the quality of the shape collapse, just the overall shape. The fact that the average absolute curvature was not near zero indicates that the avalanches did not collapse to a line. Furthermore, the curvature results indicate that sub-critical avalanches were more curved than super-critical avalanches. This result seems reasonable if we assume super-critical avalanches were longer, more sustained, and therefore flatter.

### 3.3. Dissociated cultures

In the supplemental, we present an example power-law fit, shape collapse, complexity calculation, and sub-sampling analysis of the culture data (Figures S3–S7).

**Figure 8:**
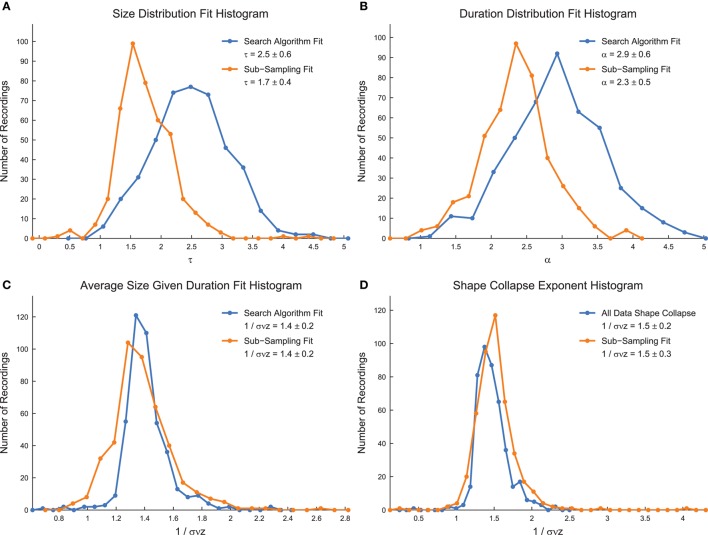
**Power-law exponents in culture data**. **(A)** Size distribution critical exponent (τ) values for all culture recordings analyzed. **(B)** Duration distribution critical exponent (α) values for all culture recordings analyzed. **(C)** Average size given duration data critical exponent (1/σ*νz*) values for all culture recordings analyzed. **(D)** Shape collapse critical exponent (1/σ*νz*) values for all culture recordings analyzed. In all subfigures, the quoted critical exponent value is mean ± standard deviation. Also, histogram bin sizes optimized using methods established in Terrell and Scott (1985).

We calculated the critical exponents using the MLE power-law search algorithm (for size distributions, duration distributions, and average size given duration data), using the full shape collapse analysis, and using sub-sampling methods (Figure 8) (Marshall et al., 2016). We found lower values of τ and α using the sub-sampling analysis, as well as lower errors (Figures 8A,B). The values of 1/σ*νz* showed little change between sub-sampling and the analysis of the full system (Figures 8C,D). The exponents found using the sub-sampling method agreed best with previously reported values for the critical exponents in neural avalanches (Mazzoni et al., 2007; Pasquale et al., 2008; Friedman et al., 2012). Note that the values of 1/σ*νz* found using distinct methods (average size given duration fits and shape collapse) agree within error. Furthermore, note that the critical exponents satisfy the general relationship given in Equation (4) using both the search algorithm and the sub-sampling method. However, we also found that randomized data satisfied the critical exponent relationship, though with different exponent values (data not shown). This result casts doubt on the importance of the critical exponent relationship as a marker for critical systems.

**Figure 9:**
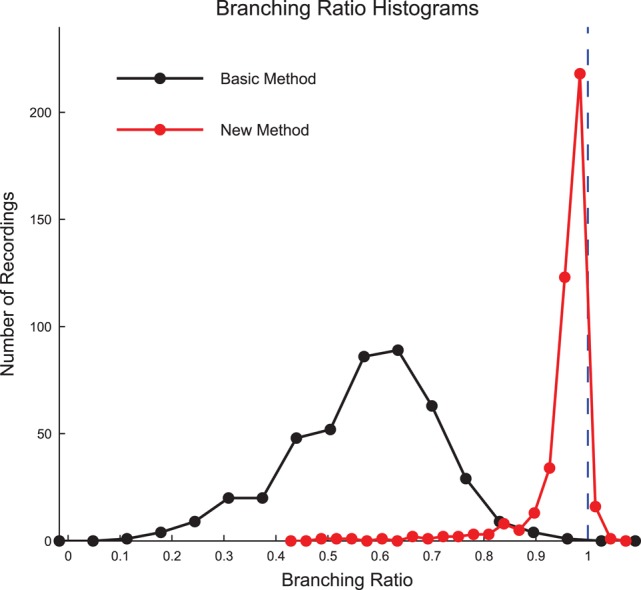
**Culture data branching ratios were near 1 after sub-sampling correction**. The branching ratios of the culture recordings were found to be close to 1 after correcting for sub-sampling using the new method established by Wilting and Priesemann (2016) (Equation 13), with most data sets being slightly sub-critical. The basic branching ratio calculation method (Equation 11) produced branching ratios that were more widely varied and strongly sub-critical. Histogram bin sizes optimized using methods established in Terrell and Scott (1985).

We calculated the branching ratio for the culture recording data using both the basic method and new method introduced by Wilting and Priesemann (2016) (Figure 9, see Section 2.11). Using the new method, we found most recordings produced branching ratios slightly below 1 (i.e., slightly sub-critical). Using the previous basic method, we found most recordings produced branching ratios that were substantially less than 1 and much more varied. The result that the majority of the data sets produced branching ratios near 1 is strong evidence that the cultures were operating near a critical point.

**Figure 10:**
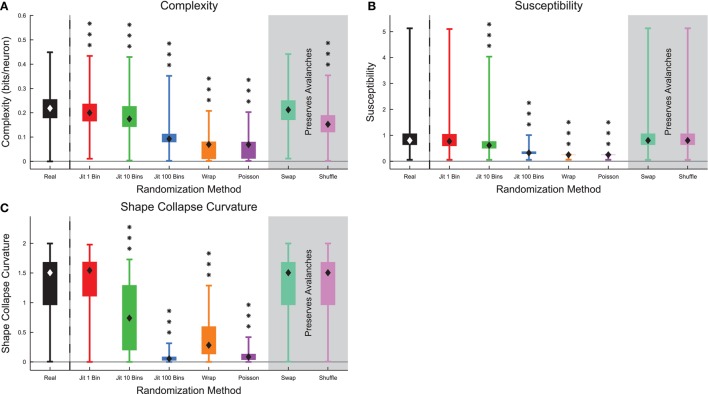
**Markers of criticality decreased under randomization in culture data**. **(A)** Complexity decreased under randomization, with the exception of spike swapping (see **Figure 12**). **(B)** The susceptibility decreased under randomization. **(C)** Avalanche shape collapse curvature decreased under randomization, indicating that the shape collapses became flatter. Note that the susceptibility and shape collapse curvature were not expected to change under spike swapping or shuffling because those randomization methods preserved avalanche profiles. Box Plots: minimum value, 25th percentile, median, 75th percentile, maximum value. Rank Sum Test: (*p* < 0.05) 1 star, (*p* < 0.01) 2 stars, and (*p* < 0.001) 3 stars. Multiple comparisons correction performed using false discovery rate control (Benjamini and Hochberg, 1995; Benjamini and Yekutieli, 2001; Groppe et al., 2011).

When we compared complexity and several markers of criticality in the full data sets (see Section 2.10) between randomized culture data and real culture data, we found that all metrics decreased substantially after randomization (Figure 10). The complexity and susceptibility decreased most under the strongest forms of randomization (e.g., Poisson randomization) (Figures 10A,B). Notably, the complexity did not decrease under spike swapping (see below). We also found a correlation of 0.23 between the complexity and the susceptibility of the recordings (see Figure S8). The shape collapse curvature decreased under randomization indicating that avalanches shape collapses became flatter (Figure 10C). In total, these results are consistent with the hypothesis that these neural systems were operating at or near a critical point and that complexity is maximized near the critical point.

**Figure 11:**
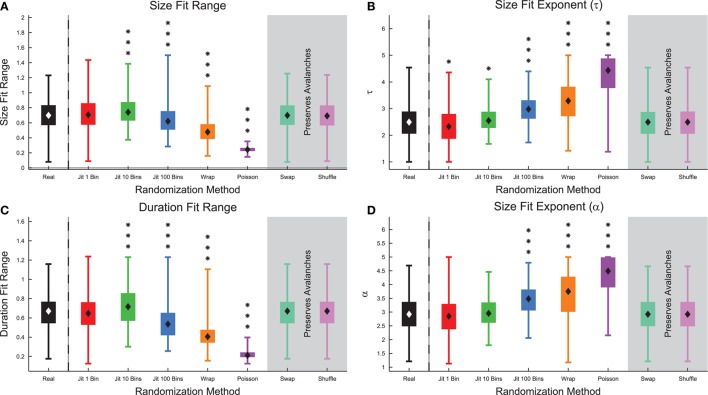
**Markers of criticality decreased under randomization in culture data**. **(A)** The size power-law fit range decreased under randomization, with the exception of the 10 bin jittering. **(B)** The size power-law fit exponent τ increased under randomization indicating the size distribution became steeper. **(C)** The duration power-law fit range decreased under randomization, with the exception of the 10 bin jittering. **(D)** The duration power-law fit exponent α increased under randomization indicating the duration distribution became steeper. Note that the fit results were not expected to change under spike swapping or shuffling because those randomization methods preserved avalanche profiles. Box Plots: minimum value, 25th percentile, median, 75th percentile, maximum value. Rank Sum Test: (*p* < 0.05) 1 star, (*p* < 0.01) 2 stars, and (*p* < 0.001) 3 stars. Multiple comparisons correction performed using false discovery rate control (Benjamini and Hochberg, 1995; Benjamini and Yekutieli, 2001; Groppe et al., 2011).

Next, we examined the size and duration fit ranges, as well as the associated fit exponents measured using all of the data (Figure 11) (see Section 2.10). We found that the fit ranges decreased for strong forms of randomization (e.g., Poisson randomization), but showed slight increases for 10 bin jittering (Figures 11A,C). We believe this may be due to errors associated with determining the characteristic time scale for the system. We found a correlation of 0.296 between the complexity and the size fit ranges of the recordings and a correlation of 0.197 between the complexity and the duration fit ranges of the recordings (see Figure S9). We also found that the power-law fit exponents increased under randomization because randomization tends to remove large or long avalanches and produce a shorter and steeper distribution of avalanche sizes or durations (Figures 11B,D).

**Figure 12:**
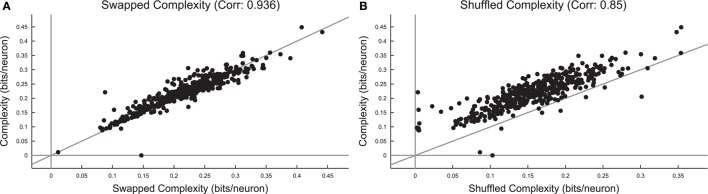
**Complexity is independent of precise spiking relationships in culture data, but dependent on firing rate**. **(A)** Comparison of complexity in real data and complexity in spike swapped randomized data for each culture data set. Note the strong correlation between the complexity values and the fact that most cultures fell near the equality line. This indicates that spiking relationships (which are disrupted to some extent by spike swapping) did not strongly affect complexity. **(B)** Comparison of complexity in real data and complexity in shuffled data for each culture data set. Note that the complexity values were strongly correlated, but complexity in the real data tended to be greater than the complexity in the shuffled data.

Surprisingly, we found that the complexity did not change substantially under spike swapping (Figure 12A), but the complexity did decrease somewhat under shuffling (Figure 12B). Furthermore, we found strong correlations between complexity in the real data and complexity in the spike swapped data (Figure 12A), as well as between complexity in the real data and the complexity in the shuffled data (Figure 12B). Recall that spike swapping preserves avalanche profiles and individual firing rates, but disrupts neuron/spike pairing to some extent, this result implies that precise spiking relationships between neurons did not strongly contribute to complexity in these neural systems. The fact that complexity decreased under shuffling indicates that neuron firing rates (which are not preserved by shuffling) did affect complexity in these neural systems.

## 4. Discussion

### 4.1. Main findings

In this work, we have produced evidence that complexity and criticality are related in neural systems. We found that complexity in a critical model was maximized near the critical point for the model. We found branching ratio values that indicated the cultures were operating near a critical point. We found critical exponents that agreed well with previous studies and expected values, as well as markers of criticality (shape collapse, power-law fitting, and susceptibility) that were not preserved under randomization. Furthermore, we found that complexity tracked well with these established markers of criticality in the culture data indicating that complexity is maximized near the critical point. Finally, we found that the complexity was primarily dependent upon neuron firing rate and avalanche profiles, but not precise spiking relationships between neurons.

### 4.2. Complexity and criticality

The hypothesis that complexity and criticality are related in neural systems seems intuitive. Systems that operate at the critical point contain correlations that range across all scales. Similarly, complexity measures the strength of correlations across across all size ranges in a system. Though, as we discuss above, complexity and criticality are not trivially related. So, the evidence we produced that complexity and criticality are related in a neural system is noteworthy.

Perhaps the most surprising result of our study was that complexity in the neural data was relatively unchanged by spike swapping. Spike swapping preserved the firing rate of each neuron and the avalanche temporal profiles (i.e., the number of active neurons in each time bin), but altered the times at which specific neurons spiked. The fact that spike swapping did not substantially alter the complexity indicates that identity of the neurons spiking at any given time (and, therefore, the precise spiking relationships between neurons) were not relevant to the complexity. We found this result surprising and counter-intuitive.

We believe the answer to this question rests in the firing rates of the neurons and the avalanche profiles. Shuffling preserved avalanche temporal profiles, but it did not preserve neuron firing rate (unlike spike swapping). When we performed shuffling, the complexity of the system decreased substantially, as we had expected. Furthermore, when we Poisson randomized the data (which disrupted avalanche profiles, but preserved individual neuron firing rates), the complexity of the system decreased substantially, as we had expected as well. Therefore, we found that avalanche profiles and neuron firing rates must be preserved to maintain complexity, but precise spiking relationships need not be preserved. We believe these requirements can be understood as follows. The avalanche profiles contain time bins with many more spikes than would be expected by chance. Furthermore, some neurons possessed many more spikes than other neurons (recall, the firing rate distribution spanned several orders of magnitude). So, even under spike swapping, it was more likely than chance that two neurons with high firing rates would fire during time bins with large amounts of activity. So, though spike swapping randomized which neurons spiked in any given time bin, the avalanche profiles and the firing rates of the neurons still imposed structure on the spiking relationships. Thus, complexity was still present. That having been said, we did not expect to find complexity values essentially unchanged by spike swapping.

We can express these results in terms of entropy using the definition of neural complexity (Equations 9 and 10). Spike swapping preserved individual neuron entropy by preserving individual neuron firing rates. Also, by preserving the avalanche profiles, spike swapping preserved the joint entropy terms in Equation (9) to some extent. Shuffling did not preserve individual neuron entropy because it does not preserve individual neuron firing rates, though it did preserve joint entropy values to some extent by preserving avalanche profiles. Poisson randomization preserved individual neuron entropy because it preserved individual neuron firing rates, but it did not preserve joint entropy values because it did not preserve avalanche profiles. Therefore, we see that both individual neuron and joint entropy values are necessary to preserve complexity.

These results provide the interesting conclusion that complexity in neural systems is driven by neuron firing rates and avalanche profiles. We believe this result should be further investigated. In particular, we hope to conduct additional studies in other neural systems to see if this result holds universally, or if it may be due to the particular circumstances of our analysis.

### 4.3. Limitations of this analysis

While our study represents a significant advance in many respects, there are three noteworthy improvements that can be made in future studies. First, we used the network-wide interspike interval (ISI) to rebin the recordings prior to our analysis. While we feel the ISI is a reasonable estimate for the characteristic time scale of the system that scales well with network size and neuron firing rates, perhaps better estimates could be found. In the future, we hope to investigate other rebinning and analysis methods to see if bin size significantly affects the results reported herein.

Second, our use of neuronal cultures allowed for large amount of data to be gathered, which greatly strengthened the statistics of the analysis. However, we used an array with only 60 electrodes and large electrode spacing. In the future we hope to analyze data from larger and more dense electrode arrays (e.g., Litke et al., 2004; Ito et al., 2014; Timme et al., 2014b, 2016a). Furthermore, *in vivo* studies of neural criticality are possible (e.g., Priesemann et al., 2009, 2014; Hahn et al., 2010; Shew et al., 2015). In the future, we hope to analyze *in vivo* data to see if the relationships between criticality and complexity we found in this analysis are found in those systems as well. Those studies would also permit research into possible relationships between criticality, complexity, and phenomena that can only be studied *in vivo*, such as behavior.

Third, one means of testing the criticality hypothesis in neural systems that we did not investigate here is attempting to tune the system through sub-critical, critical, and super-critical states (Beggs and Timme, 2012). From a thermodynamics perspective, the critical point occurs at a phase transition. In the case of a neural system, the critical point would occur at a transition between ordered and disordered phases. So, in a conceptually similar experiment to adjusting the temperature and pressure of water to tune it through liquid and gas phases to find its critical point, it may be possible to tune a neural system through different phases to locate the critical point and test the criticality hypothesis. Recent research has indicated that balanced inhibition and excitation control the critical state the neural system (Shew et al., 2009, 2011). In this work we did not carry out this type of experimental manipulation in the culture data. We did carry out a similar manipulation in a branching model. In that model, we showed that complexity peaked near the critical point and we thereby demonstrated an important connection between criticality and complexity. In the future, we hope to conduct experiments similar to those in Shew et al. (2009, 2011) to see if complexity also peaks near the critical point in a real biological system.

## Author contributions

NT and NM planned the experiment, gathered the culture data, performed the analysis, and wrote the manuscript. NB, MR, and EL gathered culture data, contributed to the analysis, and contributed to the manuscript. JB oversaw the project, planned the experiment, and contributed to the manuscript.

## Funding

This research was supported by National Science Foundation (http://www.nsf.gov) grants 090813 (JB), 1058291 (JB), CNS-0521433 (Indiana University computing resources), and CNS-0723054 (Indiana University computing resources). This research was also supported by National Institutes of Health (http://www.nih.gov) grant T32-AA007462 (NMT). This research was also supported by the Mind Science Foundation (JB). Also, via the use of computing resources at Indiana University, this research was supported in part by Lilly Endowment, Inc., through its support for the Indiana University Pervasive Technology Institute, and in part by the Indiana METACyt Initiative. The funders had no role in study design, data collection and analysis, decision to publish, or preparation of the manuscript.

### Conflict of interest statement

The authors declare that the research was conducted in the absence of any commercial or financial relationships that could be construed as a potential conflict of interest.
