# Supplementary material for: Criticality Maximizes Complexity in Neural Tissue
Source: Front Physiol. 2016 Sep 27;7:425. doi: 10.3389/fphys.2016.00425 (PMC5037237; doi:10.3389/fphys.2016.00425)
Supplement: Supplementary file 1 [file DataSheet1.PDF]

Supplementary Material: Criticality maximizes complexity in neural tissue

Nicholas M. Timme *, **Najja Marshall** *, **Nicholas Bennett, Monica Ripp, Edward Lautzenhiser, and John M. Beggs**

*Correspondence:
Nicholas M. Timme
nicholas.m.timme@gmail.com

Najja Marshall: njm2149@cumc.columbia.edu

1 CORTICAL BRANCHING MODEL

We performed sub-sampling on the complexity, susceptibility, and shape collapse curvature for the cortical branching models (Figure 1). The fitting algorithm for the complexity required a special search algorithm (see main text Section 2.10) due to discontinuous behavior in the complexity values (Figure 1 A). Note that the linear fits capture the general trends of the sub-sample values.

To assess the role played by finite recording lengths on the complexity calculation, we performed the complexity analyses discussed in the main text on cortical branching models that were run for $3 * 10^5$ (see main text for full results), $1 * 10^5$, and $3 * 10^4$ time steps (Figure 2). We found that longer recordings tended to produce higher complexity values. Furthermore, though a secondary peak in the complexity was apparent for high transmission values (similar to results shown in the main text), this peak decreased in magnitude relative to the primary peak near $p_{trans} \approx 0.28$ as the recording length increased. This analysis was identical to the analysis discussed in the main text, with the exception that all time bins were analyzed instead of only the avalanches. This control was necessary because higher transmission probability models produced longer avalanches, which would bias the complexity values higher.

2 DISSOCIATED CULTURE RESULTS

Avalanches in the culture recordings exhibited power-law behavior that was fit with the truncated power-law MLE methods Marshall et al. (2016). In the example distributions shown in Figure 3, we can see that randomizing the data tended to move the tails of the distributions lower, as expected. Similarly, the average size given duration distributions exhibited power-laws, but randomization of the data resulted in lower fit exponents (Example: Figure 4).

In addition to power-law analyses, we also employed an automated shape collapse algorithm to find the critical exponent that best collapses avalanche profiles Marshall et al. (2016). In the example shown in Figure 5 we see that the avalanche profiles in the original data were well collapsed for many profiles (Figure 5 A), as well as for three chosen profiles (Figure 5 B). When the data were randomized, a shape collapse is still possible, but the avalanches became flatter (curvature tended towards 0) and the critical exponent tended towards 1. The fact that shape collapse is still possible in randomized data further emphasizes the

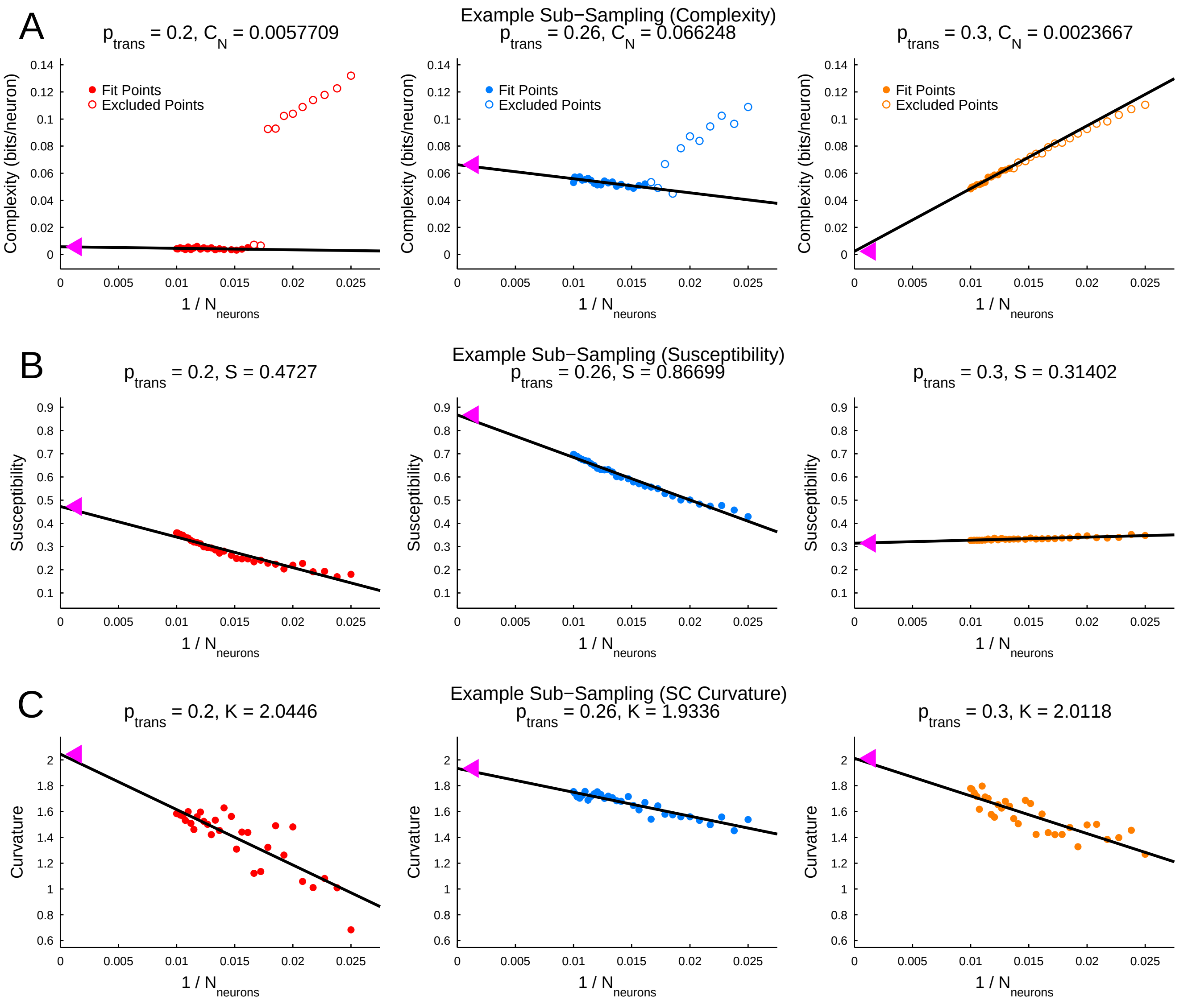

Figure 1. Cortical branching model example complexity, susceptibility, and shape collapse curvature sub-sampling. In all rows, the left column is from a sub-critical ($p_{trans} < p_{crit}$) model, the center column is from a near critical ($p_{trans} \approx p_{crit}$) model, and the right column is from a super-critical ($p_{trans} > p_{crit}$) model. Also, pink arrows represent extrapolated estimates for quantity values in an infinite system. **(A)** Complexity values. The complexity sub-sampling fits required a search algorithm to fit large sub-sample sizes due to discontinuous behavior for small transmission probabilities. **(B)** Susceptibility values. **(C)** Shape collapse curvature.

need for an automated shape collapse method and quantitative descriptions of shape collapses in terms of critical exponents and collapse shape structure (e.g. curvature).

Beyond calculating the critical exponents in the culture data as described above, we also utilized sub-sampling to estimate the critical exponents in the culture data for systems of infinite or very large size. In the example shown in Figure 6 we see that the trends in sub-sampled critical exponents were well fit using a linear function. Furthermore, the extrapolated values of $1/\sigma\nu z$ for large or infinite systems are very close for the average size given duration analysis and for the shape collapse analysis.

Finally, we calculated the complexity for the neurons found in the culture recordings. The complexity calculation utilized randomized data to correct for sub-sampling and, in some cases, applied a sub-sampling cut to the data Marshall et al. (2016). We present an example recording that did not require a sub-sampling

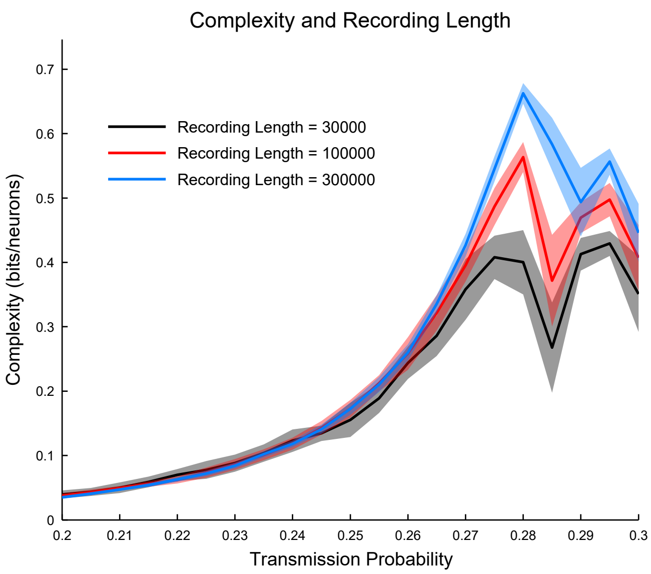

Figure 2. Cortical branching model recording length affected complexity. Complexity as a function of transmission probability for cortical branching models with three different recording lengths. Longer recordings produced higher complexity values. Furthermore, longer recordings reduced the magnitude of the peak in complexity for high transmission probability relative to the primary peak near $p_{trans} \approx 0.28$. Avalanches and silent time steps were analyzed to control for longer avalanches at high transmission probabilities. Solid line: mean complexity value across 30 models, fringe: one standard deviation of complexity values across 30 models.

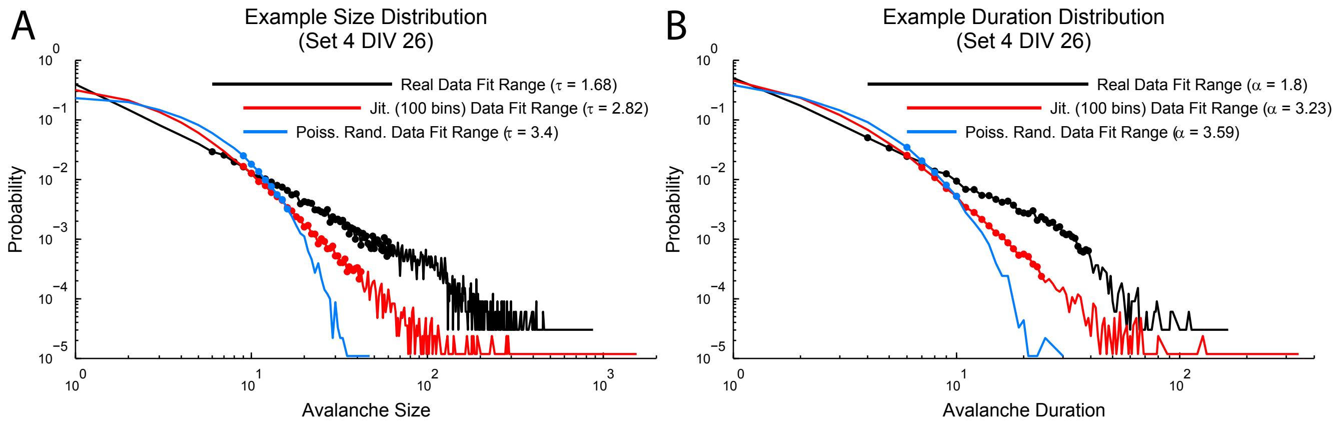

Figure 3. Example size and duration distributions from culture data. Fit regions are noted by horizontal bars and dots on the histograms.**(A)** Example size distribution. **(B)** Example duration distribution. Note that large segments of the original data are well fit by a truncated power-law and that under randomization the tails of the distributions moved downwards and the fit exponents increased.

cut after correcting for the randomized data (Figure 7 A and B), as well as an example recording that did require a cut (Figure 7 C and D). Note that in both cases, the integration for the randomized data was substantially far from 0 when considering the full system size (Figure 7 A and C). The integration should be 0 for randomized data, so these results indicate the presence of sub-sampling. We corrected the real integration curves by subtracting the integration curves from the randomized data (Figure 7 B and D). However, in some cases, the corrected integration curves became concave down (Figure 7 D), thus necessitating a cut to only consider the complexity for a smaller number of neurons that could be more adequately sampled.

We also calculated the correlation between the complexity and the susceptibility of the dissociated culture recordings (Figure 8). Finally, we calculated the correlation between the complexity and the size and

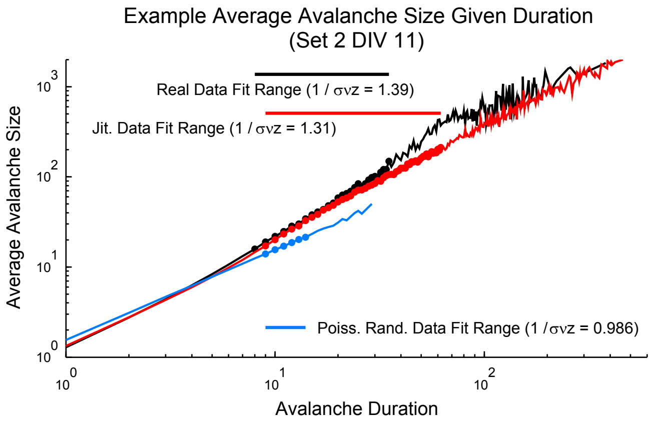

Figure 4. Example average size given duration plot from culture data. Fit regions are noted by horizontal bars and dots on the histograms. The fit regions were set by the MLE truncated power-law fit search algorithm in the duration distribution for this example data set (data not shown). The fit of the average size given duration was performed using a weighted least squares fitting (weights were the number of avalanches of each duration). Note that the real data and the randomized data exhibit power-law behavior, but that the fit exponent decreased with randomization.

duration fit ranges (Figure 9). In all three cases, we found positive correlations indicating that higher complexity data sets tended to have higher susceptibility and tended to be fit as power-laws over longer ranges.

REFERENCES

Marshall, N., Timme, N. M., Bennett, N., Ripp, M., Lautzenhiser, E., and Beggs, J. M. (2016). Analysis of power laws, shape collapses, and neural complexity: new techniques and matlab support via the ncc toolbox. *Frontiers in Physiology* 7, 250

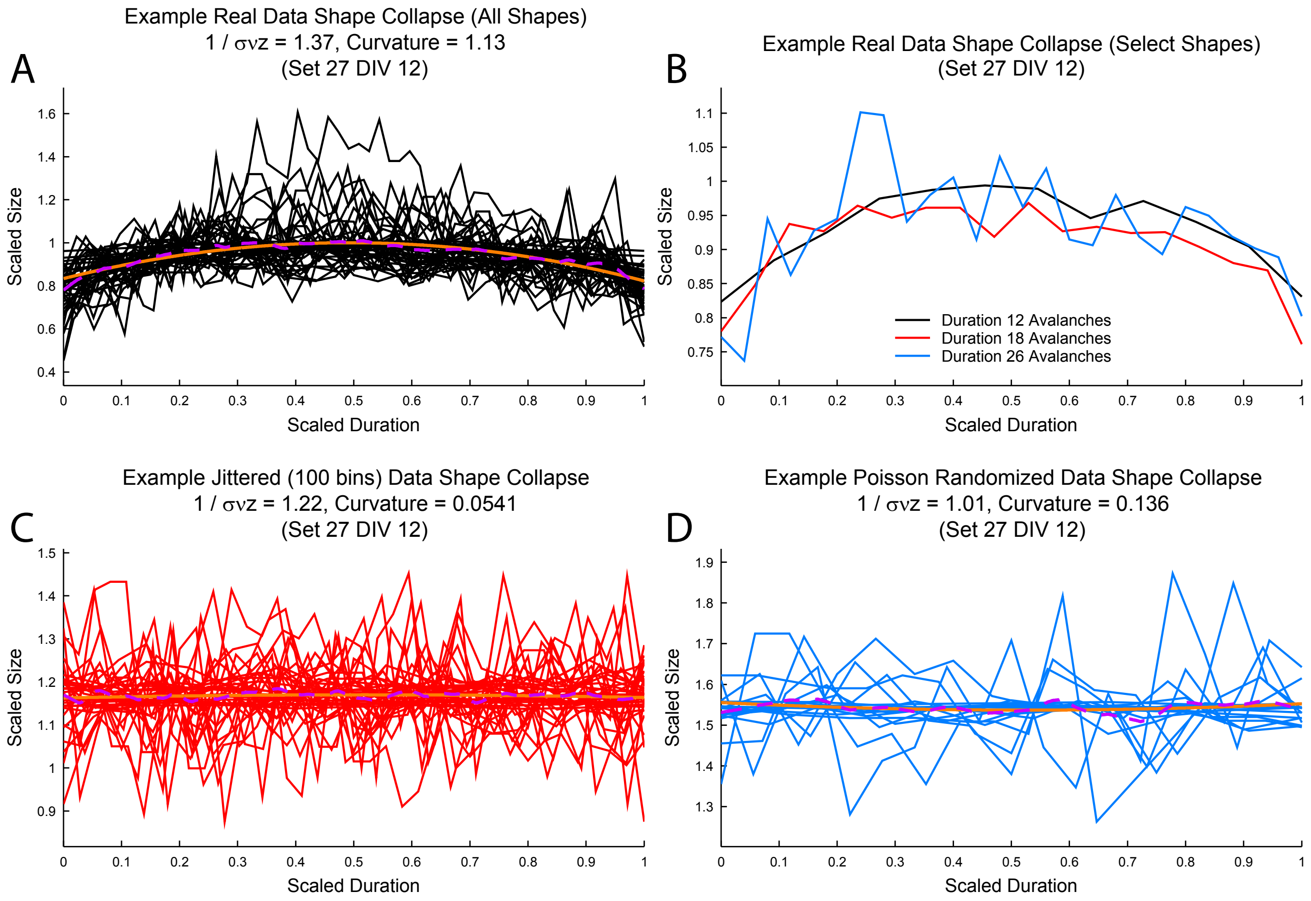

Figure 5. Example shape collapse in culture data. (A) Full shape collapse for culture data. Note the critical exponent ($1/\sigma\nu z$) value near 1.5 and the curvature value above 1. All avalanches of duration 4 or larger with at least 20 occurrences in the recording were analyzed for this shape collapse. **(B)** Three chosen avalanche profiles from (A) that more clearly demonstrate the shape collapse result. **(C and D)** Shape collapse in randomized data. Note that both avalanches showed collapse, but the avalanches were flat (curvature near 0) and the critical exponent ($1/\sigma\nu z$) for these collapses was near 1.

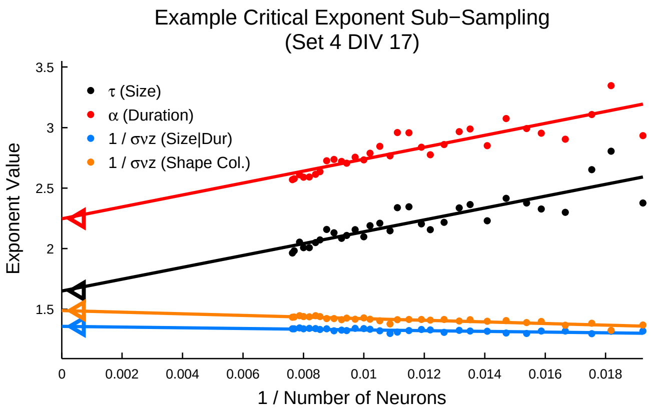

Figure 6. Example exponent sub-sampling in culture data. Note that the critical exponents from the sub-sampled systems were well fit by a linear function. Also, note the similarity between the value of $1/\sigma\nu z$ produced by the average size given duration and shape collapse methodologies.

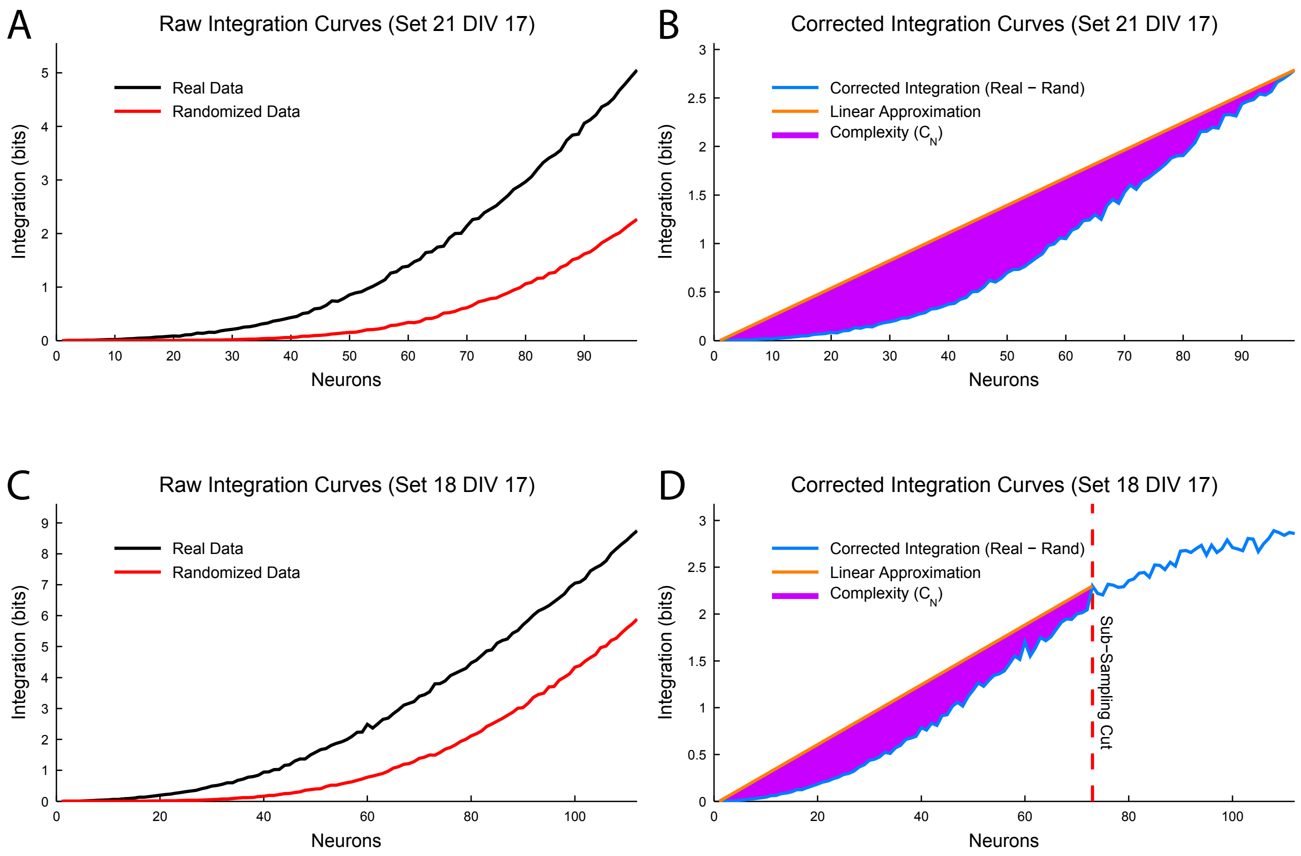

Figure 7. Example complexity calculations in culture data. (A and C) Real data and randomized data integration curves. The non-zero randomized data integration curves indicates the presence of state sub-sampling bias. **(B and D)** The corrected integration, as well as the complexity. In (B), no additional sub-sampling cut was necessary. However, in (D) the corrected integration curve became concave down, thus necessitating a sub-sampling cut.

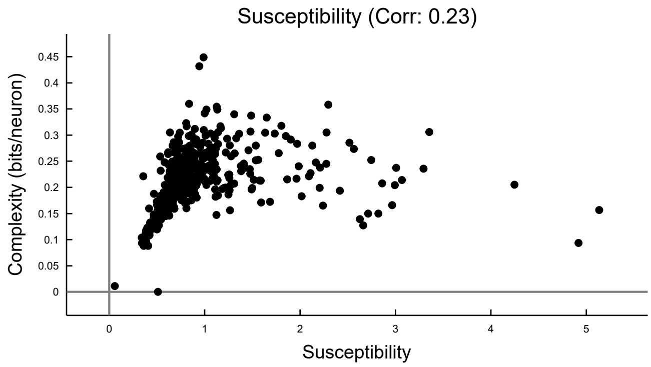

Figure 8. Complexity and susceptibility were correlated. The complexity and susceptibility of culture recordings were correlated. The relationship between the complexity and susceptibility was strongest for small susceptibility values, while for larger susceptibility values the complexity appeared to decrease.

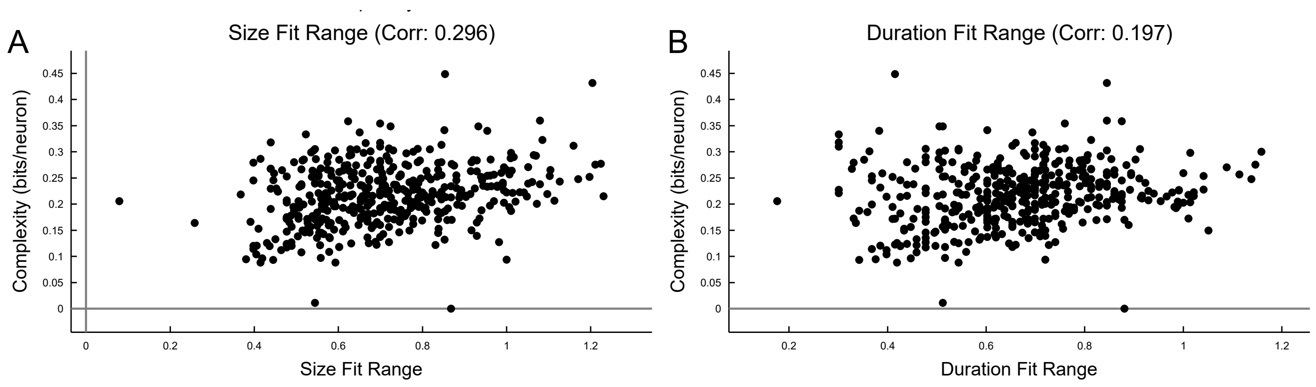

Figure 9. Complexity and power-law fit range were correlated. **(A)** Complexity and size power-law fit range were found to be correlated. **(B)** Complexity and duration power-law fit ranger were also found to be correlated.
